# The Interplay between Human Cytomegalovirus and Pathogen Recognition Receptor Signaling

**DOI:** 10.3390/v10100514

**Published:** 2018-09-20

**Authors:** Mariana Marques, Ana Rita Ferreira, Daniela Ribeiro

**Affiliations:** Institute of Biomedicine—iBiMED—and Department of Medical Sciences, University of Aveiro, 3810-193 Aveiro, Portugal; mar.marques@ua.pt (M.M.); arferreira@ua.pt (A.R.F.)

**Keywords:** human cytomegalovirus (HCMV), antiviral innate immunity, immune evasion, pattern-recognition receptors (PRRs), pathogen-associated molecular patterns (PAMPs)

## Abstract

The cellular antiviral innate immune response is triggered upon recognition of specific viral components by a set of the host’s cytoplasmic or membrane-bound receptors. This interaction induces specific signaling cascades that culminate with the production of interferons and the expression of interferon-stimulated genes and pro-inflammatory cytokines that act as antiviral factors, suppressing viral replication and restricting infection. Here, we review and discuss the different mechanisms by which each of these receptors is able to recognize and signal infection by the human cytomegalovirus (HCMV), an important human pathogen mainly associated with severe brain defects in newborns and disabilities in immunocompromised individuals. We further present and discuss the many sophisticated strategies developed by HCMV to evade these different signaling mechanisms and counteract the cellular antiviral response, in order to support cell viability and sustain its slow replication cycle.

## 1. Introduction

The human cytomegalovirus (HCMV), a species-specific *β-herpesvirus*, is a highly widespread opportunistic pathogen that infects people of all ages, with higher seroprevalence in the elderly [1]. In a healthy immunocompetent host, primary HCMV infection is almost always benign with minimal or no clinical manifestations, although it can result in horizontal or vertical transmission, and can occasionally cause a self-limited mononucleosis syndrome, sore throat, glandular fever, or mild hepatitis [2]. However, in immature or compromised immune subsets of the population (including patients who are undergoing hemodialysis or receiving immunosuppressive drugs, and patients with cancer or, infected with human immunodeficiency virus (HIV), or organ transplant recipients), it may lead to serious illness, culminating in organ damage and life-threatening diseases [3,4]. HCMV was also implicated in age-related diseases such as vascular pathologies [5], and was pinpointed as one of the major causes of congenital disorder, leading to severe and permanent neurological injury in newborns [6].

HCMV is the largest of the eight known human herpesviruses. Its long non-segmented linear double-stranded (ds) deoxyribonucleic acid (DNA) genome encodes for many proteins and micro ribonucleic acids (miRNAs) [7], and is surrounded by a symmetric icosahedral capsid which is, in turn, enclosed into a lipid bilayer spiked with glycoprotein complexes [8]. The tegument, an amorphous matrix, is localized in between these two layers and holds cellular and viral ribonucleic acids (RNAs), as well as the majority of the virion proteins [9].

HCMV infects and replicates in a remarkably extensive variety of human cells (reviewed in Reference [10]), going through two distinct phases, a productive lytic phase and a life-long non-productive latent phase, where the virus remains silent in the host with periodically productive reactivation. Overall, this contributes to HCMV’s efficient systemic spread and transmission [11,12].

Attachment and fusion of infectious particles with the host cell membrane requires interaction of several viral glycoproteins, e.g., gB and gH [13,14], with cell-surface proteoglycans and receptors [15,16]. After internalization, virion RNAs are translated in the cytoplasm and viral capsids are transported into the nucleus, where viral transcription, genome replication, and encapsidation occur [17]. At late times post infection, the viral capsids assemble in nuclear viral factories, and associate with the tegument at the cytoplasm; finally, enveloped infectious particles are released via exocytosis to spread the infection [18].

The innate immune system is the first line of defense against pathogens and plays a major role in restricting infection against viruses. The cellular antiviral immunity is activated with the recognition of pathogen-associated molecular patterns (PAMPs) by a set of the host’s membrane-bound or cytoplasmic pattern-recognition receptors (PRRs), inducing the early production of interferons (IFNs), and the expression of IFN-stimulated genes (ISGs) and pro-inflammatory cytokines. These suppress viral replication and restrict infection through the activation and nuclear translocation of nuclear factor kappa B (NF-κB), IFN-regulatory factor 3 (IRF3), and IRF7, culminating in the expression of a variety of innate immune genes (reviewed in References [19,20,21]). However, viruses adopt numerous and specific evasion strategies that help subverting these host immune responses.

Following HCMV infection, a strong NF-κB-dependent production of type I IFNs, ISGs, and pro-inflammatory cytokines is rapidly induced, establishing an antiviral response within the cell, as well as in the neighboring cells [22,23]. Having a slow replication cycle, HCMV depends on sustained cell viability, and, to prevent the premature death of infected cells, the virus is able to block apoptotic signaling pathways and developed highly sophisticated immune evasion strategies that allow efficiently manipulation of the immune system. In this review, we discuss the different mechanisms of PRR-mediated HCMV immune-sensing by the infected cells, and furthermore, we extend our discussion to the most recent findings on the strategies developed by this virus to counteract and efficiently suppress the cellular innate immune response upon virion recognition.

## 2. Toll-Like Receptors in HCMV Infection

Toll-like receptors (TLRs) play a crucial role in non-specific immunity and are considered the primary pathogen sensors [24]. Each TLR can specifically recognize different pathogen structures: TLR2 and TLR4 seem to be generally involved in the recognition of viral proteins [25,26], while TLR3, TLR7/8, and TLR9 are respectively linked to the recognition of double stranded RNA (dsRNA), single-stranded RNA (ssRNA), and unmethylated cytosine–phosphate–guanine (CpG) motifs in viral DNA in different cellular subsets [25,27,28].

**Figure 1:**
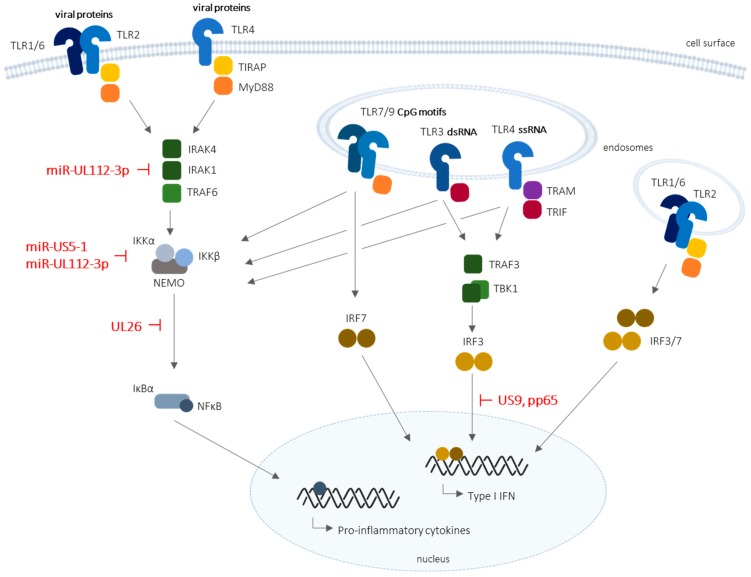
Schematic representation of Toll-like receptor (TLR) signaling and corresponding evasion mechanisms developed by the human cytomegalovirus (HCMV). TLR2 and TLR4 are present on the cell surface, and are directly stimulated or internalized upon activation, whereas TLR3 and TLR7/8 are localized in intracellular endosomes. HCMV microRNAs (miR-UL112-3p and miRUS5-1), as well as HCMV (UL26, US9, and pp65) proteins, are known to inhibit specific or broad antiviral mechanisms downstream of TLR recognition, in particular nuclear factor kappa B (NF-κB) and interferon (IFN) regulatory factor 3 (IRF3)-mediated type I IFN production. IκBα: NF-κB inhibitor; IKK: IκB kinase; IRAK: interleukin-1 receptor-associated kinase; MyD88: myeloid differentiation primary response 88; NEMO: NF-κB essential modulator; TRAF: tumor necrosis factor (TNF) receptor-associated factor 1; TBK1: TRAF family member-associated NF-κB activator (TANK)-binding kinase protein 1; TRAM: translocating chain-associated membrane protein; TRIF: Toll/interleukin receptor (TIR)-domain-containing adapter-inducing IFN-β. T-bars represent inhibitory actions.

Activation of TLRs stimulates the upregulation of inflammatory cytokines and type I IFNs. As detailed in Figure 1, upon HCMV infection, TLR stimulation results in the activation of several transcription factors, including NF-κB and activator protein 1 (AP-1), which govern the expression of inflammatory cytokines, IRF3 and IRF7, regulating the activation of IFN-mediated responses [29].

As there is no animal model that can efficiently be infected by HCMV, most studies are performed in human cell cultures, sometimes complemented by ex vivo tissue models that likely more accurately represent in vivo infections. Studies performed on human cell lines showed that TLR2 directly interacts with HCMV gB and gH glycoproteins, leading to an NF-κB-mediated upregulation of inflammatory cytokines, such as interleukins 6 and 8 (IL-6 and IL-8), but not IFNs [26]. These results, together with others [30,31], reinforce TLR2 function in the initiation of an inflammatory cytokine response against HCMV infection. However, Juckem et al. [32] suggested that the IFN response to HCMV is not an endosomal TLR2-dependent process, but instead, relies on cholesterol-rich microdomains.

TLR2, TRL3, and TLR9 were determined as inducing the expressions of IFN-β and tumor necrosis factor alpha (TNF-α) at early times during HCMV infection in human THP-1 cells, as well as in human foreskin fibroblasts (HFF) [30]. However, other studies demonstrated that, in human monocyte-derived dendritic cells (moDCs), the early HCMV-triggered immune response appears to be independent of TLR3 signaling [33].

Additionally, Harwani et al. [34] showed that distinct TLR ligands inhibit HCMV infection by inducing IFN-β production in HFF and ectocervical explants. Both TLR3 and TLR4 were correlated with the inhibition of HCMV infection in HFF and cervical tissue, while TRL2 and TLR9 also induced HCMV inhibition in ectocervical explants. These differences are likely simply due to the absence of TLR2 and TLR9 from HFF cell cultures, emphasizing the importance of ex vivo studies to complement HMCV infection experiments in isolated cell cultures. However, further studies in clinically relevant cell culture models would surely be equally valuable.

Work done by Yew et al. (2012) and Arcangeletti et al. (2013) further demonstrates that the TLR4/lymphocyte antigen 96 (MD2)/cluster of differentiation 14 (CD14) complex contributes to HCMV-induced signaling and cytokine production in monocytes [35] and in THP-1 cells [36]. Iversen et al. [37] showed that TLR9 is upregulated in HCMV-infected fibroblasts, suggesting that TLR9-dependent signaling may be important for antiviral defense.

Recent studies demonstrate that HCMV developed specific strategies to counteract some TLR-dependent signaling mechanisms (depicted in Figure 1 and summarized in Table 1).

HCMV microRNAs miR-US5-1 and miR-UL112-3p were shown to dampen NF-κB signaling by specifically targeting NF-κB inhibitor (IκBα) kinase alpha and beta (IKKα and IKKβ) signaling factors to limit cytokine production [38]. Previously, HCMV miR-UL112-3p was found to target TLR2 innate immunity. At late times during infection, in fibroblasts and monocytic THP-1 cells, the accumulation of viral miR-UL112-3p was correlated with a decrease in TLR2 protein level. This activity was shown to be modulated by the inhibition of both interleukin-1 receptor-associated kinase (IRAK1) and NF-κB, and a reduction in cytokine expression [39].

## 3. Cytosolic DNA Sensors in HCMV Infection

**Figure 2:**
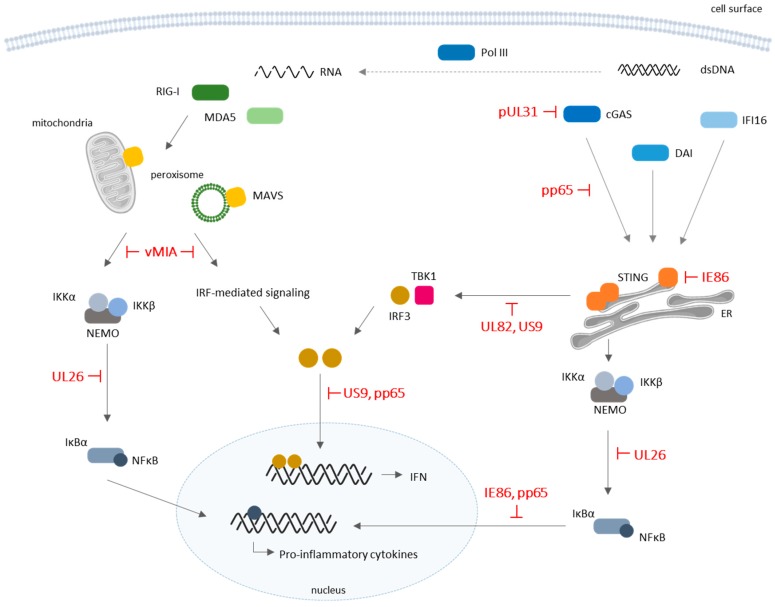
Schematic representation of cytosolic pattern-recognition receptor (PRR) signaling and corresponding evasion mechanisms by HCMV. Several cytosolic sensors are involved in the recognition of viral DNA and RNA and downstream activation of IFNs and pro-inflammatory cytokines production with antiviral functions. HCMV proteins were shown to modulate the host’s innate immune system by dampening cytosolic PRR-mediated signaling. Along with broader modulation of antiviral IFNs and cytokine production, HCMV seems to act effectively on retinoic acid-inducible gene I (RIG-I)/mitochondrial antiviral-signaling protein (MAVS) and cyclic guanosine monophosphate–adenosine monophosphate (cGAMP) synthase (cGAS)/endoplasmic-reticulum (ER) protein stimulator of interferon genes (STING) pathways. DAI: DNA-dependent activator of IFN-regulatory factors, also known as Z-DNA-binding protein 1 (ZBP1); dsDNA: double-stranded deoxyribonucleic acid; IFI16: IFN-γ inducible protein 16; IRF: IFN regulatory factor; MDA5: melanoma differentiation-associated protein 5; Pol III: RNA polymerase III. T-bars represent inhibitor actions.

In general, cytosolic viral genome recognition promotes the expression and secretion of IFNs which, in turn, drive the expression of ISGs, that antagonize viral replication and protect uninfected cells from subsequent infections. There are multiple types of cytosolic DNA-sensing PRRs: cyclic guanosine monophosphate–adenosine monophosphate (cGAMP) synthase (cGAS), DNA-dependent RNA polymerase III (Pol III), absent in melanoma 2 (AIM2), IFN-γ-inducible protein 16 (IFI16), and the DNA-dependent activator of IFN-regulatory factors (DAI) (Figure 2) (reviewed in References [40,41]).

cGAS is considered as the main intracellular DNA sensor involved in the activation of innate immune responses against DNA viruses. Upon recognition and binding to viral nucleic acids, cGAS catalyzes the synthesis of cGAMP which, in turn, binds to and activates the endoplasmic-reticulum (ER) protein stimulator of interferon genes (STING) to induce IRF3-mediated type I IFN production [42,43]. Interestingly, in virus-producing cells, cGAS-synthesized cGAMP can be packaged in viral particles and extracellular vesicles that efficiently deliver it to uninfected target cells, to propagate and activate antiviral immune responses in those cells [44]. Once active, STING dimerizes and acts as a scaffold protein to promote the phosphorylation of IRF3 by TNF receptor-associated factor (TRAF) family member-associated NF-κB activator (TANK)-binding kinase 1 (TBK1), leading to its activation and downstream production of type I IFNs [45,46,47]. Furthermore, the STING/TBK1 complex interacts with the NF-κB inhibitor (IκB) kinase (IKK) complex to activate and induce the translocation of NF-κB into the nucleus, promoting the expression of pro-inflammatory cytokines [48].

HCMV infection rapidly induces the activation of IRF3 leading to the expression of IFNs and ISGs [22,49,50]. The cGAS/STING/IRF3 signaling axis was demonstrated by Lio et al. [51] to be essential to mediate the initial TLR-independent antiviral responses against CMV, with the production of a robust amount of type I IFNs that further limits early CMV replication, either in primary human endothelial cells or mice.

Paijo et al. [52] suggested that HCMV-infected monocyte-derived cells synthesize abundant cGAMP levels that precede type I IFN production, establishing cGAS as a key sensor of HCMV-mediated type I IFN induction in primary human moDCs and macrophages. However, the same authors found that, despite constitutively expressing cGAS, plasmacytoid DCs (pDCs) induce type I IFN responses in a TLR9-dependent manner.

AIM2-mediated inflammasome [53], IFI16 [54,55], and DAI (or Z-DNA-binding protein 1-ZBP-1) [56] were shown to detect HCMV and to be involved in the host defense against infection, binding viral DNA and triggering expression of antiviral cytokines. AIM2 and IFI16 receptors induce the inflammasome after recognizing intracellular dsDNA [57], and IFI16 further activates a STING-dependent signaling pathway [58]. It was reported that interactions between the HCMV tegument pUL83 (pp65) protein and AIM2 disrupted the activation of AIM2 and reduced the AIM2 inflammasome-associated proteins [59]. The same viral protein was also shown to interact with the IFI16 sensor [55,60].

DAI has, likewise, an integral role in the DNA-mediated induction of type I IFNs and other genes involved in innate immunity via its association with IRF3 and TBK1 [61]. In HCMV-infected human fibroblasts, DAI was shown to be essential for the induction of the expression of IFN-β, mediated by the DAI-dependent activation of STING and IRF3 [56].

Many strategies were already unraveled by which HCMV is able to evade the DNA sensor-dependent antiviral signaling (depicted in Figure 2 and summarized in Table 1).

Browne et al. [62] demonstrated that fibroblast infection with a mutant virus lacking the major viral structural protein pUL83 (pp65) caused a stronger induction of many IFN responses and pro-inflammatory chemokine RNAs than infection with the wild-type virus. The authors argued that this protein was downregulating the virion-induced IFN responses by directly impacting NF-κB. However, a different study by Abate et al. demonstrated that pp65 may constitute a viral evasion factor to counteract the antiviral response, as it modulates the rapid induction of an IFN-like response through the inhibition of IRF3 activation rather than NF-κB [63]. Additionally, using a mutant HCMV unable to express UL83-encoded pp65, Biolatti et al. [64] showed that this protein might be involved in dampening IFN-β production in HFF cells, as it selectively binds to cGAS early during infection and prevents its interaction with STING, thus inactivating the cGAS/STING/IRF3 signaling axis.

The HCMV UL31 protein was, likewise, identified as an inhibitor of cGAS. This protein can directly interact with cGAS and inhibit its enzymatic activity, thereby reducing cGAMP production and downstream antiviral gene expression. UL31 overexpression was shown to markedly contribute to HCMV replication in HFF cells, suggesting that this protein is involved in the evasion of the innate response to HCMV [65].

Other studies suggest that, during infection, HCMV appears to inhibit STING-mediated signaling to evade the immune response. For instance, Fu et al. [66] identified the tegument protein UL82 as a negative regulator of STING-dependent antiviral response, as it impairs the assembly of the STING/TBK1/IRF3 complex, preventing the trafficking of STING from the ER to perinuclear microsomes. Also, the UL122-encoded immediate–early 86 kDa (IE86) protein strongly abolishes cGAMP-mediated type I IFN promoter activation, as it post-translationally regulates STING, to enable its proteasome-dependent degradation, and inhibits cGAMP-mediated induction of IFNs and chemokine C–X–C motif ligand 1 (CXCL1), an antiviral cytokine [67]. Taylor et al. [68,69] previously suggested that, during HCMV infection, IE86 blocks the expression of cytokines, namely IFN-β and pro-inflammatory chemokines, by acting as an NF-κB antagonist.

Choi et al. [70] recently showed that the HCMV-encoded US9 glycoprotein inhibits IFN-β production and antiviral responses by targeting STING-mediated signaling in late stages of HCMV infection, to evade host innate antiviral responses. US9 expression abrogated STING-mediated IRF3 nuclear translocation, by altering STING oligomerization and STING/TBK1 disruption at the ER. Overall, US9 leads to IRF3 cytosolic sequestration, thus inhibiting IRF3 nuclear translocation and IFN-β production.

Other proteins were also correlated with HCMV innate immune evasion. In particular, Mathers et al. [71] found that HCMV UL26 protein antagonizes (TNF-α-induced) NF-κB activation by attenuating the phosphorylation of IKK. However, and as other herpesviruses, HCMV appears to target NF-κB signaling differentially throughout the course of infection, as its prolonged activation seems to be crucial for an efficient replication [72]. For instance, the HCMV UL144 protein was also shown to be a potent activator of NF-κB-induced transcription, through a mechanism dependent on TRAF6 and tripartite motif 23 (TRIM23) proteins, involved in TLR-mediated signaling, in HCMV-permissive human cells [73,74].

## 4. Is HCMV Antiviral Signaling Triggered by RNA Sensors?

RNA sensors, such as the family of retinoic acid-inducible gene I (RIG-I)-like receptors (RLRs), which include RIG-I and melanoma differentiation-associated protein 5 (MDA5) (reviewed in References [40,41]), recognize viral RNA in the cytoplasm of infected cells and induce the production of inflammatory cytokines and type I IFNs (Figure 2). Structurally, RLRs contain an intermediate RNA helicase domain, which is involved in recognition and binding to pathogen nucleic acids, a C-terminal repressor domain, and two N-terminal tandem caspase activation and recruitment domains (CARDs) [75,76,77]. Upon viral infection, the recognition and binding of exogenous RNA structures leads to a conformational switch of RIG-I, which releases the auto-repressed CARDs [78,79] and recruits its downstream adaptor mitochondrial antiviral-signaling protein (MAVS) at mitochondria [80] and peroxisomes [81]. In contrast to RIG-I, MDA5 has its CARD domains permanently exposed [82]. MAVS activation induces the formation of detergent-resistant prion fiber-like active aggregates, which are essential for the activation of downstream signaling pathways [83]. Both peroxisomal and mitochondrial MAVS have different but complementing activities: peroxisomal MAVS was shown to be involved in early but transient responses, while mitochondrial MAVS seems to act with slower kinetics, inducing delayed but long-lasting responses [81,84].

The RLR/MAVS and cGAS/STING pathways mediate antiviral responses against RNA and DNA virus infections, respectively. However, an interplay between these two pathways, as well as the possible consequent involvement of both signaling mechanisms in antiviral immunity against RNA and DNA viruses, was already suggested. In fact, the downstream signaling components of both pathways are physically and functionally interconnected. For instance, STING was reported to influence IFN expression during RNA virus infection [85,86,87,88], acting as a cofactor in the RIG-I-mediated IFN response to RNA viruses, as it interacts with both RIG-I and MAVS. Recently, Liu et al. [89] demonstrate, both in vitro and in vivo, that the activation of the RIG-I pathway, with an RNA agonist, can cease a DNA virus infection, through NF-κB and signal transducer and activator of transcription 1/2 (STAT1/2) synergy, mediated by STING-dependent TNF-α and IFN-α production. With these results, the authors corroborated the crosstalk between the RIG-I/MAVS RNA-sensing and cGAS/STING DNA-sensing pathways. However, other reports show contradictory results, indicating that STING does not regulate RNA-induced IFN expression. Upon stimulation with polyinosinic–polycytidylic acid (poly(I:C)) or infection with the Sendai virus, a dsRNA analog and an RNA virus, respectively, IFN-β induction was not affected in the absence of cGAS or STING [43]. Recently, a study performed by Franz et al. verified that STING was not necessary to induce IFN expression in RNA-virus-infected fibroblasts; however, it was still required to restrict its replication [90].

The stimulation of the RLR/MAVS signaling pathway by DNA viruses was also already demonstrated. A recent study suggested that dsDNA, either synthetic or pathogen-derived, can activate RLR-mediated innate immune responses through an IRF3-dependent type I IFN gene induction [91]. One other report showed that one of the early recognition mechanisms of herpes simplex virus (HSV) occurs through MDA5 sensing in human primary macrophages, which is linked to a virus-induced IFN response [92]. Another dsDNA virus, the Epstein–Barr virus (EBV) was also shown to induce type I IFN responses mediated by RIG-I, NF-κB, and IRF3, since RIG-I can sense EBV-encoded small RNAs [93].

Interestingly, other groups demonstrated that cytosolic dsDNA can be sensed by Pol III and converted into an RIG-I-recognizable RNA form to activate RIG-I and induce downstream antiviral innate immune responses, including IFN and NF-κB activation [94,95].

To the best of our knowledge, there are yet no studies specifically reporting the recognition of HCMV by RLRs. However, it is known that cellular dsRNA accumulates during HCMV infection and can trigger antiviral innate immune mechanisms against it. To counteract this response, HCMV encodes for IRS1 and TRS1, dsRNA-binding proteins that can prevent and evade dsRNA-activated antiviral pathways, namely the protein kinase R (PKR)-mediated response [96]. It was also demonstrated that the HCMV-encoded viral mitochondria-localized inhibitor of apoptosis (vMIA) protein [97,98] is able to induce mitochondrial fragmentation and inhibit the mitochondria-dependent signaling pathway downstream from MAVS [99]. vMIA was also shown to localize at peroxisomes, where it interacts with MAVS and specifically inhibits the peroxisomal MAVS-dependent antiviral response [100]. One other report demonstrated the degradation of RIG-I during HCMV infection [101]. Furthermore, the HCMV-encoded US9 glycoprotein was shown to target MAVS-mediated signaling and inhibit IFN-β production in later stages of infection [70]. Altogether, these results seem to point to a possible involvement of the RLR-dependent mechanisms in antiviral signaling against HCMV.

## 5. Concluding Remarks

As many other viruses, HCMV is recognized by a variety of receptors from the host cell, which signal its presence and induce the production of different compounds, impairing virus particle production and inhibiting viral spread. HCMV’s PAMPs interact with a variety of members from the TLR family such as TLR2, TLR3, TLR4, and TLR9, and are also recognized by the cytoplasmic sensors cGAS, AIM2, IFI16, and DAI. Recent results also suggest that, similar to other DNA viruses, HCMV may also be signaled via RLR sensors, although further studies are needed to confirm this hypothesis. With such a variety of cellular antiviral sensors and signaling mechanisms, it is quite remarkable that the virus is still able to maintain cell viability and sustain a slow replication cycle. This is mainly due to its remarkable capacity to prevent multiple cell-death pathways and evade many of the antiviral responses, including the PRR-dependent signaling pathways. Table 1 summarizes the distinct mechanisms involving HCMV proteins and microRNAs that are used by the virus to efficiently subvert the membrane and cytoplasmic sensor signaling pathways that were reviewed and discussed in this manuscript.

Unraveling the mechanisms developed by HCMV to interact with the host cell and evade the innate immune response mechanisms not only enhances our knowledge of the viral pathogenesis, but also provides insight into the mechanisms of the cellular innate immunity. Further developments might contribute to the discovery of novel improved treatments against this virus, which still causes significant morbidity and mortality in neonatal and immunocompromised patients.

## Figures and Tables

**Table 1:** Human cytomegalovirus (HCMV) evasion from the antiviral innate immune responses.

Viral Factor	Function	Cell Type (Strain)	Reference
miR-US5-1	Targets IKKα and IKKβ to limit production of pro-inflammatory cytokines	NHDF cells (TB40/E)	[38]
miR-UL112-3p	Targets IKKα and IKKβ to limit production of pro-inflammatory cytokines	NHDF cells (TB40/E)	[38]
	Targets and downregulates TLR2 and inhibits its dependent activation of IRAK1 and NF-κB signaling	NHDF cells (AD169) THP-1 (TB40E)	[39]
pUL83 (pp65)	Inhibits IFN-α and antiviral gene expression by blocking IRF1 and NF-κB activity	HFF (AD169)	[62]
	Modulates the rapid induction of an IFN-like response by inhibiting IRF3 activation	HFF (AD169)	[63]
	Dampens IFN-β production by selectively binding to cGAS, inactivating the cGAS/STING/IRF3 axis	HFF (TB40E)	[64]
pUL31	Downregulates antiviral gene expression by directly interacting with cGAS	HEK293T, HFF (AD169)	[65]
pUL82	Prevents STING trafficking to the ER and impairs the formation of TBK1/IRF3/STING complexes	HEK293T, HFF, MLF (AD169)	[66]
pUL122 (IE86)	Mediates proteasome-dependent STING degradation and inhibits cellular transcription factors for IFN-β promoter activation	HFF (Towne)	[67]
	NF-κB antagonist; suppresses NF-κB-dependent cytokine and chemokine gene expression	MRC5 fibroblasts (AD169)	[68]
US9	Inhibits IFN-β production and antiviral responses by targeting both MAVS- and STING-mediated signaling	HEK293T, HFF (AD169)	[70]
pUL26	Antagonizes NF-κB activation by attenuating IKK phosphorylation	MRC5 (AD169)	[71]
pUL144	Agonist of NF-κB-induced transcription via TRAF6 and TRIM23	U373, HFF (AD169, TB40E)	[73,74]
vMIA (pUL37 × 1)	Inhibits mitochondrial MAVS-dependent antiviral signaling	HeLa (transfection)	[99]
	Inhibits the peroxisomal MAVS-dependent antiviral signaling	HepG2, HFF, Mefs (transfection)	[100]

cGAS: cyclic guanosine monophosphate–adenosine monophosphate synthase; ER: endoplasmic reticulum; IFN: interferon; IKK: NF-κB inhibitor (IκBα) kinase; IRAK: interleukin-1 receptor-associated kinase; IRF: IFN regulatory factor; MAVS: mitochondrial antiviral-signaling protein; NF-κB (nuclear factor kappa B); STING: stimulator of IFN genes; TBK1: TRAF family member-associated NF-κB activator (TANK)-binding kinase protein 1; TLR: Tool-like receptor; TRAF: tumor necrosis factor (TNF) receptor-associated factor; TRIM: Toll/interleukin receptor (TIR)-domain-containing adapter-inducing IFN-β.
